# Perceptions of vaginal microbicides as an HIV prevention method among health care providers in KwaZulu-Natal, South Africa

**DOI:** 10.1186/1742-6405-4-7

**Published:** 2007-03-14

**Authors:** Gita Ramjee, Neetha S Morar, James Mtimkulu, Joanne E Mantell, Varanna Gharbaharan

**Affiliations:** 1 South African Medical Research Council, HIV Prevention Research Unit, 123 Jan Hofmeyer Road, Westville, 3630, Durban, South Africa; 2 HIV Center for Clinical & Behavioral Studies, at the New York State Psychiatric Institute and Columbia University, 1051 Riverside Drive, Unit 15, New York, NY 10032, USA; 3 Mailman School of Public Health, Columbia University, Department of Population and Family Health, New York, NY 10032, USA; 4 Nelson R. Mandela School of Medicine, University of KwaZulu-Natal, Durban, South Africa

## Abstract

**Background:**

The promise of microbicides as an HIV prevention method will not be realized if not supported by health care providers. They are the primary source of sexual health information for potential users, in both the public and private health sectors. Therefore, the aim of this study was to determine perceptions of vaginal microbicides as a potential HIV prevention method among health care providers in Durban and Hlabisa, South Africa, using a combination of quantitative and qualitative methods.

**Results:**

During 2004, semi structured interviews with 149 health care providers were conducted. Fifty seven percent of hospital managers, 40% of pharmacists and 35% of nurses possessed some basic knowledge of microbicides, such as the product being used intra-vaginally before sex to prevent HIV infection. The majority of them were positive about microbicides and were willing to counsel users regarding potential use. Providers from both public and private sectors felt that an effective microbicide should be available to all people, regardless of HIV status. Providers felt that the product should be accessed over-the-counter in pharmacies and in retail stores. They also felt a need for potential microbicides to be available free of charge, and packaged with clear instructions. The media was seen by health care providers as being an effective strategy for promoting microbicides.

**Conclusion:**

Overall, health care providers were very positive about the possible introduction of an effective microbicide for HIV prevention. The findings generated by this study illustrated the need for training health care providers prior to making the product accessible, as well as the importance of addressing the potential barriers to use of the product by women. These are important concerns in the health care community, and this study also served to educate them for the day when research becomes reality.

## Background

Evidence from studies of the female condom ([1-5]), emergency contraception [6], and medical abortion ([7,8]) reminds us of the potent influence that health care providers' (HCPs') beliefs and attitudes can have on the promotion of these technologies to potential users (PUs). In the field of HIV prevention, emerging technologies like microbicides have the potential to impact public health significantly, and the role that HCPs play as their patients' primary source of HIV and STI information will be crucial in successfully dispensing, educating and providing access to microbicides, once they become available [9].

Mantell and colleagues (2005) have discussed the introduction of the female condom in the early nineties, drawing the comparison with microbicides as a novel, women – initiated HIV prevention method [4,10]. The female condom has not had the impact on reducing HIV transmission that researchers had hoped for, and this is due in large part to the lack of acceptability research among HCPs prior to introduction. Most research was conducted after the female condom had been introduced, and HCPs were often unprepared to counsel and educate PUs into making informed choices regarding its use [10]. With the female condom, a lack of awareness among HCPs regarding design features, cost as well as unfamiliarity with various physical characteristics of the product, also contributed to low acceptability among PUs [10].

Drawing on the lessons learnt from the female condom, researchers in the field of microbicides are now keenly aware of the importance of acceptability studies among HCPs prior to product introduction

Sub-Saharan Africa is bearing the brunt of the HIV pandemic, with women accounting for a large part of new infections. HCPs beliefs and attitudes in sub-Saharan Africa, as well as the role they play as educators, have been found to significantly influence PUs acceptance of a product [2-4]. Bearing this in mind, as well as the fact that new HIV infections occur mostly among women in this region, it becomes critical that researchers investigate HCPs level of awareness and opinions regarding the promotion of vaginal microbicides as an HIV prevention method.

HCPs' also play a key role in determining the best channels for access and distribution of novel HIV prevention and reproductive health methods[6,8,10,14]. In countries with high HIV and AIDS prevalence, public sector workload and resources are severely strained and may limit HCPs' ability to promote and market microbicides to PUs' [14]. Moreover, there has been a dearth of research on the impact of the health care delivery systems on the adoption of new disease prevention technologies and the need for adaptations in service provision. As such, it is not known how HCPs will cope with the potential introduction of a microbicide. Therefore, one of the objectives of the present study was to investigate HCPs opinions regarding channels for delivery, access and distribution.

This study conducted by the Medical Research Council of South Africas' HIV Prevention Research Unit (HPRU), represents the first comprehensive attempt to understand the views of HCPs with regard to promoting potential microbicides. The participants were not given information on microbicides prior to data collection by the interviewers. However, the study was conducted in areas where extensive education was provided to the community at large, including HCPs

## Methods

### Ethical approval

Ethical approval was obtained from the University of KwaZulu-Natal Biomedical Research Ethics Committee (BREC). Approval was obtained from the KwaZulu-Natal Provincial Department of Health to approach public hospital staff, whilst private sector health care providers were approached directly by project staff.

### Study population and setting

The study population consisted of 149 HCPs recruited from 53 clinics and hospitals. The majority of these were facilities that serviced the public sector (49/53), whilst the remainder were private hospitals (4/53). Their locations were in the city of Durban and the rural district of Hlabisa in KwaZulu-Natal Province, South Africa. All private hospitals were situated in Durban. Since the majority of the South African population access services through the public health service due to economic reasons, the researchers purposively sampled more HCPs from this sector. Clinics were randomly selected by health districts using the provincial Department of Health's list of health care centres.

**Figure 1:**
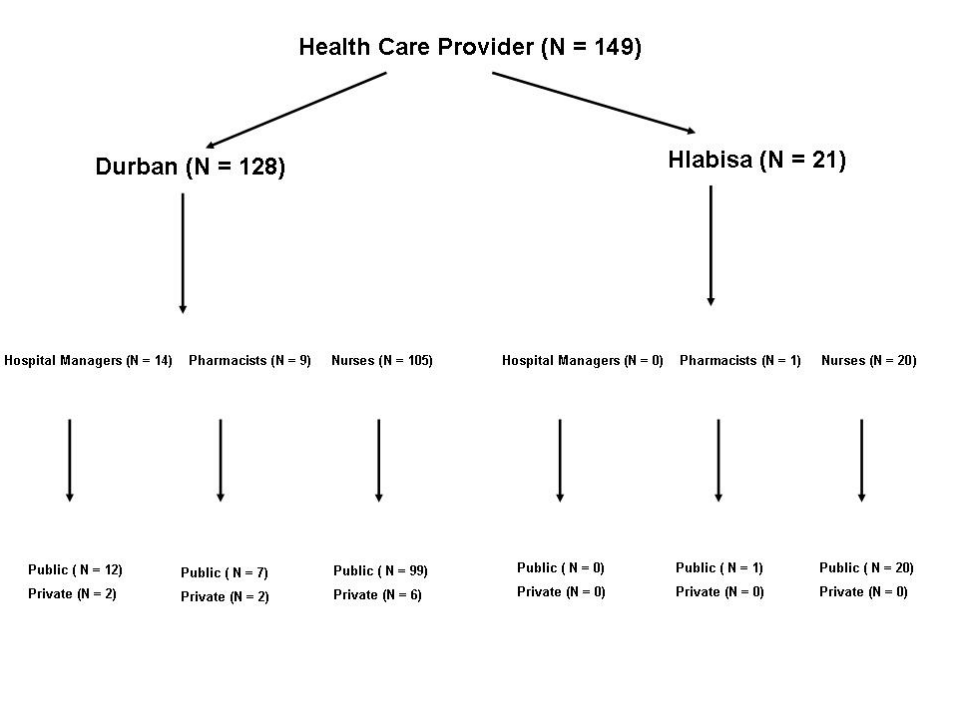
Study population and settings.

The 149 HCPs recruited consisted of 14 hospital managers (HMs), 10 pharmacists and 125 nurses. Participants were purposively sampled, and more nurses were recruited since they form the backbone of public sector health services. Physicians were not included since they do not interact with PUs to the extent that nurses do. Traditional healers were also not included in the sampling as the study was focused on HCPs from the formal health sector. Pharmacists were included since they are ideally positioned to increase people's access to microbicides, in terms of product placement and dispensing. Numerous clinics are often serviced by a single pharmacist, thereby accounting for the lower number of pharmacists sampled. Hospital Managers were recruited so as to obtain views on access and distribution, as well as capacity building needs with regard to a potential large-scale microbicide roll-out. The majority of HCPs were recruited from Durban, since Hlabisa has a limited public health service sector with only 20/125 nurses and 1/10 pharmacists being recruited from the latter area. Refer to Figure 1 that shows the population and setting breakdown.

### Data collection and procedures

Data collection for the study took place between February and November 2004. A semi-structured key informant interview (SSI) with each HCP was conducted. The SSIs consisted of both closed and open-ended questions and were used to obtain individual perspectives from the HCPs professional position.

Focus Group Discussions (FGDs) were also held with the Chief Professional Nurses (CPNs) from the local health authority. All CPNs were sampled to represent clinic areas in Durban and there were 5 FGDs with between 7 and 12 members per FGD. 90% of the participants were women, as there are few men in the nursing services in the public sector. The FGDs were aimed at understanding the group dynamics that might impact on nurses' attitudes towards potential microbicides.

At each facility, researchers met with managers and staff to discuss the project and recruitment strategy. A team member described the study purpose, procedures, audio-recording, confidentiality, and obtained informed consent. Interview appointments were thereafter scheduled with some HCPs, whilst others were interviewed at the time of study recruitment.

### Semi-structured interviews

The SSI explored four core domains: Section A dealt with *participants' socio-demographic details *(gender, area and type of practice, race, and religious affiliation).

Section B focused on *descriptions and opinions about microbicides*. This section questioned HCPs on their awareness of microbicides, source, and content of information; when information was acquired; opinion on its use for HIV prevention; target groups and age restrictions for promotion; disease prevention effectiveness relative to condoms; and intentions to recommend to others.

Section C dealt with *barriers to and facilitators of the introduction of microbicides*. HCPs were asked about cultural, political, religious and social; literacy and communication barriers between researchers and the target community; there willingness to counsel clients; challenges in promoting method in clinics and adequacy of staff resources.

Section D was concerned with *marketing strategies for microbicides*. Questions were asked regarding the most appropriate media communication channels, opinion leaders and communication agents, ideal times for promotion, preferred venues for obtaining microbicides, packaging preferences, and cost.

All HCPs were asked the same questions on demographic details in section A. For section B, questions were the same across job strata with a few exceptions:

(a) Nurses and Pharmacists were further questioned on whether they would recommend microbicides to PUs if they were 'as effective as condoms' and 'less effective than condoms'.

(b) The interviewer probed nurses and pharmacists on the question dealing with a potential microbicide that is 'less effective than condoms', by asking if they would recommend such a microbicide (i) with or without condoms; (ii) to be used more often without a condom; (iii) and alternate between condoms and microbicide.

For section C, questions were tailored to provide information on how HMs would deal with obstacles facing their hospitals, staff and clients, in the event of a large-scale microbicide roll-out. With pharmacists, questions regarding the challenges of product placement and effective dispensation were emphasized. With nurses, different questions in this theme were asked to gain insight as to how they would counsel and educate PUs about using a potential microbicide, and the challenges that may go with it.

Section D on marketing strategies asked the same questions, and was targeted towards pharmacists and nurses only.

### Focus group discussions

The FGDs were conducted among CPNs using questions similar to the open-ended questions from the SSI questionnaires. The following questions and themes were explored:

(a) Information about microbicides.

(b) Microbicides as an STI/HIV prevention method.

(c) Groups of PUs to which the microbicide should be dispensed to.

(d) Factors that affect the dispensation of microbicides in the health sector.

(e) Preferred marketing strategies in the introduction of the microbicide to the public.

(f) The most effective strategy to provide information to the clinic.

(g) If microbicides were introduced in the health care system, how would you like them to be introduced in clinics and hospitals?

(h) How should microbicides be packaged?

(i) Dispensation of the products in the pharmacy.

(j) If microbicides were to be dispensed in stores, where should it be displayed?

(k) If microbicides were introduced, how would you want PUs to obtain them?

(l) The acceptability of products to PUs.

### Data analysis

Quantitative data from the SSIs were entered into an Epi-Info™ Version 6.4D database and checked twice prior to analysis with SPSS™ version 11.5. Data frequencies and tables were prepared and content analysis of the responses to the open-ended questions in the SSIs and FGDs was performed to identify and code salient themes, which were thereafter analyzed quantitatively using SPSS™ version 11.5. During content analysis, new codes emerged inductively following reading of the data. The codes were developed independently by research staff, who held meetings to achieve consensus about the coding categories and met regularly to resolve discrepancies.

The SSIs and FGDs were conducted by bilingual Zulu-English research staff with experience in conducting qualitative and quantitative interviews. Research staff were trained by senior study staff. All interviews were audio-recorded to ensure accuracy and quality of data and were transcribed verbatim.

## Results

### (1) Socio-demographic characteristics of participants

The 149 HCPs consisted of 14 HMs, 10 pharmacists, and 125 nurses. Ninety four percent (140/149) were female and from the public sector (93%, 138/149), with 78% (116/149) being of African descent. All of the HMs, 90% (9/10) of pharmacists and 84% (105/125) of nurses were from Durban. The remaining single pharmacist and 16% (20/125) of nurses worked in Hlabisa. Most of the HCPs (87%) followed the Christian faith.

In terms of type of practice/facility, 57% (71/125) of nurses worked in primary health care clinics, whilst 28% (35/125) worked in comprehensive facilities. The remaining 15% (19/125) of nurses were spread between family planning clinics, sexually transmitted diseases clinics and the like. For pharmacists, half of them worked in a clinic environment, whilst the remaining 50% (5/10) worked in commercial pharmacies. For the HMs, 78% (11/14) worked in primary health care environments, whilst the remainder came from other hospital settings.

### (2) Health care providers descriptions and opinions about microbicides

#### Description's of microbicides

This aspect of the study was undertaken to assess HCPs ideas and beliefs about what microbicides were, including descriptions of physical features and intended purpose. Fifty-seven percent (8/14) of HMs, 40% (4/10) of pharmacists and 35% (44/125) of nurses had heard about microbicides before. Most participants had acquired this information in the previous year (2003), and primarily from the HPRU's training and community entry programmes that are run throughout the province. A broad and simple definition of microbicides was also provided on the SSI questionnaire itself as follows:

"A microbicide could be used with the male and female condom for extra protection. Some people may choose to use them without condoms. There are many factors that will impact on women's decisions to use these substances".

HMs with prior information of microbicides had a vague understanding of the candidate products, and described them as a '*cream [that] prevents STIs*; a *cream [that] prevents pregnancy*; *they will kill...microorganisms*.' However, some were able to describe microbicides in more specific terms – '*can be used as a protection against STDs even AIDS *and...*applied in the vagina by an applicator before sexual intercourse*.'

Whilst the 40% (4/10) of pharmacists with prior information of microbicides had a better understanding when compared to HMs, only one pharmacist (1/10) was aware that the active ingredient in microbicides is still unknown, given that all the products are currently in the testing phase.

Nurses who had prior information of microbicides as a potential HIV prevention method were reasonably accurate in their descriptions of candidate products – '*a gel...applied by females to prevent sexually transmitted infections*.' One nurse expressed the following unsettling view about microbicide research: *'The rumour was that they *(researchers)*will ask you to sleep with a positive person *(HIV)*to prove whether it works*.'

#### Microbicides as a prevention method and empowering tool for women

Hospital managers saw potential microbicides as an empowering tool for women, recognizing that a person could *'make a decision alone without having to involve the partner*' and that '*men are resisting using condoms'*. Pharmacists said that they were '*an excellent idea*, *convenient *and *good but not guaranteed to be used without a condom because a condom is used for more than one purpose*, here referring to the advantage of contraception that condoms have over potential microbicides, where the latter may or may not be indicated for contraception. Most nurses recognized that microbicides potentially could empower women, '*especially in our Black culture*' and corroborated the view that '*males don't want to use condoms*'. One nurse supported microbicides '*as long as *[they are] *not going to be messy'*.

Fifteen nurses were uncertain of their feelings about potential products. Only four of the 94 nurses who had prior knowledge about microbicides had negative opinions of them, challenging their potential acceptability and effectiveness. This was reflected in the following statements:

*'If *[a]*sex worker uses it, how effective is it going to be for her to carry it in her purse? Cultural beliefs may be a restriction.'*

*'I don't think it will work or...be acceptable...because I think the gel is messy'*.

*'I cannot guarantee it might prevent *[HIV]*since it's in a gel form. Gel is usually slippery'*.

*'Sexual investigations proved HIV not to be manageable. No hope at all*.'

#### Access to microbicides

Most HCPs (77%) thought that microbicides should be dispensed to sexually active people whether infected or not with HIV and other STIs. One hospital manager felt this way because 'everybody is potentially HIV-positive until proven otherwise'. One pharmacist thought that since many people do not disclose their HIV status to their sexual partners, it would be better to give everyone access to microbicides. Nurses who wanted to dispense the product to all people believed that HIV positive people should have access to microbicides to prevent re-infection, decrease HIV/STI transmission to others, and prevent the acquisition of other STIs. For non-infected people, primary prevention of HIV infection was the rationale behind the choice.

The remaining 23% of the HCPs felt that only some groups of people should receive the product when it becomes available. One HM felt that it could be detrimental to administer microbicides to those already infected because: '*if given to HIV-infected, people will have myths and mistake the product with the cure and start doing anyhow when it comes to sexual issues'*, *i.e*., sexual promiscuity may result. One pharmacist advocated microbicides for those infected with STIs – '*A STI patient is a candidate for HIV. If you do not treat, STIs, increases...chances of...HIV.'*

Whilst the majority of HCPs felt that no age restriction should be implemented when microbicides are introduced, 17% (25/149) of them believed differently. Some HCPs who supported a no restriction policy felt that adolescents should be targeted because they are '*sexual *[ly]*active with more than one partner'*. Pharmacists agreed with promoting microbicides to all people of all ages, provided that the '*generic composition of the product...is safe' *for all age groups. These HCPs had strong sentiments regarding the issue of HIV and sexual behaviour among young people :

*'HIV *[is]*not restricted to any particular age'*. [HM]

*'Cannot put age restriction because even the 12-year-olds are sexually active'*. [HM]

*'Sometimes you find a very young boy doing sex with a very young girl only to find that the condom does not fit this boy'*. [Nurse]

*'It [potential microbicide] should be given to anyone willing to use it'*. [Nurse]

The HCPs who supported age restrictions on access (17%) felt that youth might abuse microbicides and not take further precautions to prevent disease transmission. Others among this group felt that they should be reserved for adults '*because...you want to encourage abstinence for the young person'*. A pharmacist pointed out that they were '*not allowed to dispense to minors below 14 years otherwise we need informed consent*.'

#### Promoting microbicides as a partially effective prevention method, and condom use

When asked if they would recommend potential microbicides if proven to be less effective than condoms, 80% (8/10) of pharmacists and 75% (93/125) of nurses responded in the affirmative. The majority of these HCPs believed some disease protection was preferable to none. Pharmacists indicated they would recommend a microbicide which was less effective relative to a condom because '*safer sex is better than unprotected sex*;*for the safety of the female' *and because '*a microbicide is not visible'*, unlike the female condom. However, most pharmacists stated that in this case they would prefer to recommend '*both the gel and condom'*. One pharmacist who would not recommend a microbicide of partial efficacy preferred to '*improve the product so that it can have the desired effect'*. The same pharmacist did indicate, however, that a cheaper product which '*may not have the entire effect' *may still have to be recommended. Nurses, even those who responded negatively to the question, endorsed the recommendation of both methods for '*dual protection'*.

Among nurses in Hlabisa, 60% (12/20) reported that they would not recommend partially effective microbicides, whilst in Durban 19% (20/105) would not. One reason for this discrepancy was that half of the nurses in Hlabisa misunderstood "less effective" as "not effective at all", despite clarification by the interviewer. This is illustrated by the following examples:

'*It's a waste of time to recommend something ineffective'*.

*'No point in using something useless'*.

When asked if they would recommend microbicides to their clients if they were as good as condoms in preventing HIV and STIs, almost all nurses and pharmacists (~ 100%) were unanimous that they would. Some of the reasons offered were:

*'HIV is a priority these days and is threatening everyone...We are willing to use the best product that we can get'*. [Pharmacist]

*'Any drug that has positive therapeutic benefit, is a drug of choice'*. [Pharmacist]

*'Because our aim is to fight against HIV and STI's'*. [Nurse]

While agreeing to recommend them, one nurse noted that the decision to use them would be left to the client: '*In the same way that we promote condom usage, we will do the same to the gel. We will give clients the option to choose'*.

Negative perceptions of condoms and the advantages of microbicides over condoms were cited as further reasons to recommend potential microbicides of equivalent efficacy as condoms. Concerns about efficacy for pregnancy prevention, breakage, allergic reactions, and non-use were reported as impediments to condom use. In fact, 24% (30/125) of nurses mentioned the disadvantages of condoms and that their clients did not want to use them. In contrast, microbicides were seen as easy to use, providing an alternative prevention option and enhancing sexual sensation, as reflected in HCPs' comments below.

*'People find it difficult to put on condoms and *[it]*does not take time to apply anything into the vagina. A female can do it prior *[to]*...sexual intercourse. It provides protection without forcing the other partner to use a condom.' *[Pharmacist]

The one nurse who was against recommending potential microbicides of equivalent condom efficacy felt that [she]*'can only ask for a person to choose to use either of the two'*, *i.e*., in support of informed client choice.

#### Spreading the message for microbicide usage

Almost all (99%) of the 149 HCPs verbalised that their colleagues would be willing to recommend potential microbicides to clients if proven effective for HIV prevention. One HCP said that '*HIV infection is a problem. We do discuss our programmes. We evaluate our programmes. We test people at this clinic. If there are any means that...can be done to prevent this we should try it'*. Another stated that she wished to '*supply all the information so that the person takes an informed decision'*.

One pharmacist felt that 'a *nything to prevent the disease should be used. HCPs should be more knowledgeable about these. They need training so that they can spread the word around'*. Two of the 10 pharmacists indicated that '*the cost factor' *was important, microbicides should be '*economical for clients'*. Overall, a great majority of nurses would support '*anything to prevent the disease because it is a kille*r and *we see what ...HIV is doing to the patients every day'*.

All of the nurses and pharmacists reported that they were willing to counsel clients about using microbicides for HIV/STI prevention. Nurses saw counseling as their '*duty' *and '*more effective than just issuing without counseling'*, *i.e*., dispensing microbicides without providing information about them. Many nurses pointed out that since they counseled patients about condoms, they would do the same with microbicides, encouraging clients to make '*informed choices'*. One pharmacist commented: '*The more knowledgeable people are about medication, the more rationally it will be dispensed'*.

### (3) Barriers to and facilitators for the introduction of microbicides in the public health setting

#### Potential barriers

Twenty one percent (3/14) of managers, 70% (7/10) of pharmacists and 62% (76/125) of nurses anticipated various barriers to the introduction of microbicides. We classified types of barriers as political, religious, cultural, level of literacy, miscommunication between researchers and community, time, resources, training needs, and other.

Managers were concerned about service providers not being properly informed about the product, as reflected in the following statement:

'It means that before the product is introduced they would have to be informed, given lectures and it is only then that they (HCPs) may try and promote it'

One manager mentioned specific problems at his health care centre:

'*We do not have an antenatal or post natal facility nor do we have a family planning clinic*'

Presumably, a lack of such facilities would act as an obstacle to microbicide delivery. Other concerns that HMs had are reflected in these quotes below:

Pharmacists worried about the following issues:

*'Tendering government pharmaceutical stores. If you haven't got the government system, it will delay the process'*.

*'Prescribers may not want to prescribe if the demand is too high'*.

*'A person may never anticipate when he/she is going to have sex'*.

Pharmacists also noted that microbicides might be problematic for users and partners who prefer inserting intra-vaginal substances for dry sex.

Among nurses, many indicated that men, especially among those who are of African descent, would not condone women taking control over sexual matters. One social barrier noted was that '*the public may be skeptical' *about microbicides. Some nurses felt that educating people about microbicides would be difficult if they were not provided with '*enough information'*.

Many nurses cited a shortage of staff and limited space as barriers. '*Nurses' attitude *[s]*towards microbicides...if...negative' *also was perceived to be an impediment. Cost was perceived to be a potential barrier, with some participants anticipating that microbicides will cost more than condoms.

Nurses were further asked about possible challenges in promoting microbicides to patients in the clinics. In addition to cost and limited staff resources, other challenges noted were limited availability and sustainability of product in clinics; potential user embarrassment (e.g., '*It might be difficult to demonstrate the use of this product'*), shyness, and/or discomfort in using a new method; lack of information and knowledge about the product, particularly regarding effectiveness; cultural myths; beliefs about product efficacy (e.g., '*The people...thinking that this product will cure HIV') *and male partners' reactions (e.g., '*If we are giving it to females, we don't know how her partner will react')*.

However, many nurses did not anticipate any challenges to microbicide introduction in clinics – and believed '*that the patients will be happy to have access to this product'*.

#### Resources for distribution

Among nurses and pharmacists, nearly all (97%) felt that their facilities were properly situated for microbicide distribution. Those who disagreed were probed regarding what could be done to improve access – '*Usage of mobile clinics *and *Teams (health workers) doing home visits' *were suggested. Among the HM's, 29% (4/14) felt that their staff would be sufficient to handle the demands of product roll-out. To ensure adequate staff resources for this programme, the common sentiment among managers was that they would '*have to motivate for more staff from the department'*. One manager felt that '*government should provide more staff whenever introducing new product [s]' *and that '*enough space to accommodate clients' *must be provided.

Hospital managers's were asked how they would introduce the new product to their staff. Most opted for in-service training workshops; whilst one manager suggested that '*somebody from the company that deals with the product should come and train staff'*. When asked how they would introduce clients to microbicides, managers recommended advertising and health education – '*Everybody who comes to the clinic should be informed'*.

#### Counseling for microbicide use

Nurses and pharmacists were asked to rate how effective certain groups and venues would be for counseling clients and promoting a potential microbicide. Ninety percent (9/10) of pharmacists felt clinics would be highly effective. When asked about chemists, schools, hospitals and NGOs, 60% (6/10) of pharmacists thought that these groups would be highly effective. Forty percent (4/10) of pharmacists, however, were uncertain about the role traditional healers could play in counseling. Sixty percent (75/125) of nurses felt clinics would be a highly effective or effective venue for counseling users about microbicides, and a similar proportion (57%, 71/125) felt the same about hospitals.

### (4) Marketing strategies for microbicides

#### Promotion venues

Nurses and pharmacists were asked to rate the effectiveness of various venues for marketing microbicides, including advertising *via *radio, newspapers, TV, leaflets, posters, taxi ranks, billboards, and retail outlets. Although most pharmacists considered all of the abovementioned strategies to be highly effective, they rated leaflets, taxi ranks, and retail outlets as less effective marketing strategies. About three-quarters of the nurses rated TV and radio advertising to be highly effective. Similarly, like the pharmacists, while each type of advertising was evaluated as highly effective by some nurses, advertising on billboards, in taxi ranks and retail outlets was viewed to be the least effective strategies. Nearly two-fifths (59%) of nurses felt that TV advertisements promoting microbicides should be screened during all hours of the day. Forty percent (4/10) of pharmacists agreed. However, another 40% (4/10) of the pharmacists and about 22% (28/125) of nurses felt that the most appropriate time for these promotions would be in the evenings. In terms of radio advertisements, similar proportions of pharmacists (80%) (8/10) and 77% (96/125) of nurses agreed that advertisements on radio promoting microbicides should be screened all of the time.

#### Promotion strategies

Nurses and pharmacists were asked how they would want microbicides to be promoted in hospitals and clinics and were given the following response options: family planning programmes, one-to-one counseling by nurses, advertisements on posters in doctors' rooms, life orientation programmes by clinics in schools, and leaflets in clinics. Eighty percent (8/10) of pharmacists and 87% (109/125) of nurses opted for all of the above.

#### Over-the-counter dispensing in pharmacies and retail stores

**Figure 2:**
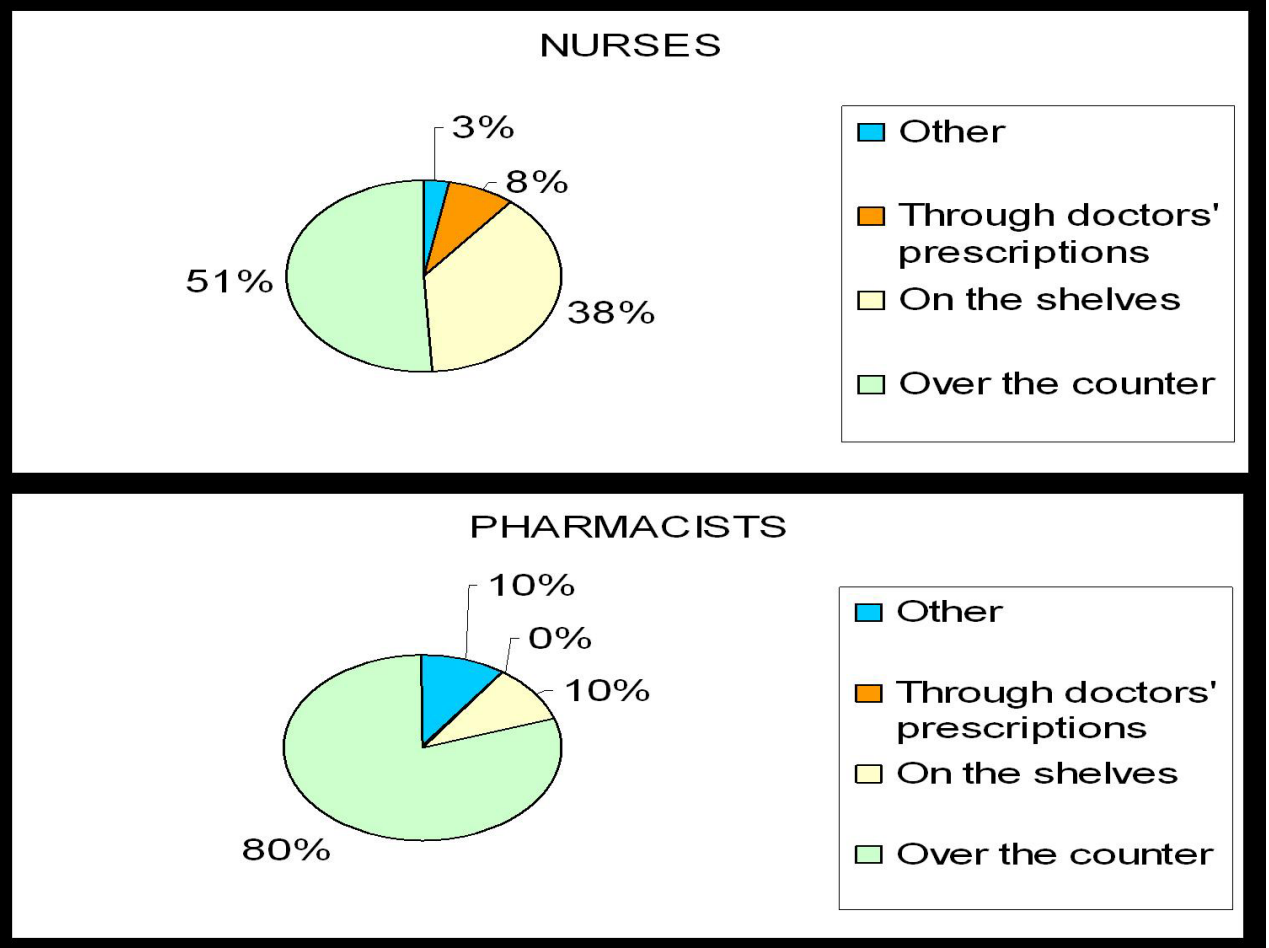
Preferences regarding dispensing of microbicides among pharmacists and nurses.

As shown in Figure 2, the majority of pharmacists (80%, 8/10) and nurses (51%, 64/125) would like microbicides to be available over-the-counter. Over-the-counter refers here to products being placed behind store/pharmacy counters, separate from being placed on shelves. In order for the product to be obtained from behind a counter, it would have to be requested for, whereas product placement on shelves can be anonymously retrieved without requesting help from any store/pharmacy attendant. Pharmacists and nurses who preferred over-the-counter dispensing were against doctors' prescriptions due to the added cost of a consultation fee and the frequent lack of availability of doctors. In addition, over-the-counter was preferred for '*counseling purposes – Advice on side effects and how to use the product' *can be given. One pharmacist had this to say: '*Maybe...everyone will be too shy to go and get it although this one seems like an expensive product so maybe over-the-counter'*.

Some nurses opted for doctors to prescribe the product because they felt that '*the doctor will explain to you how to use them'*. Those who preferred the product to be placed on the shelves preferred this '*so that people will not be embarrassed ...asking for the gel'*.

**Figure 3:**
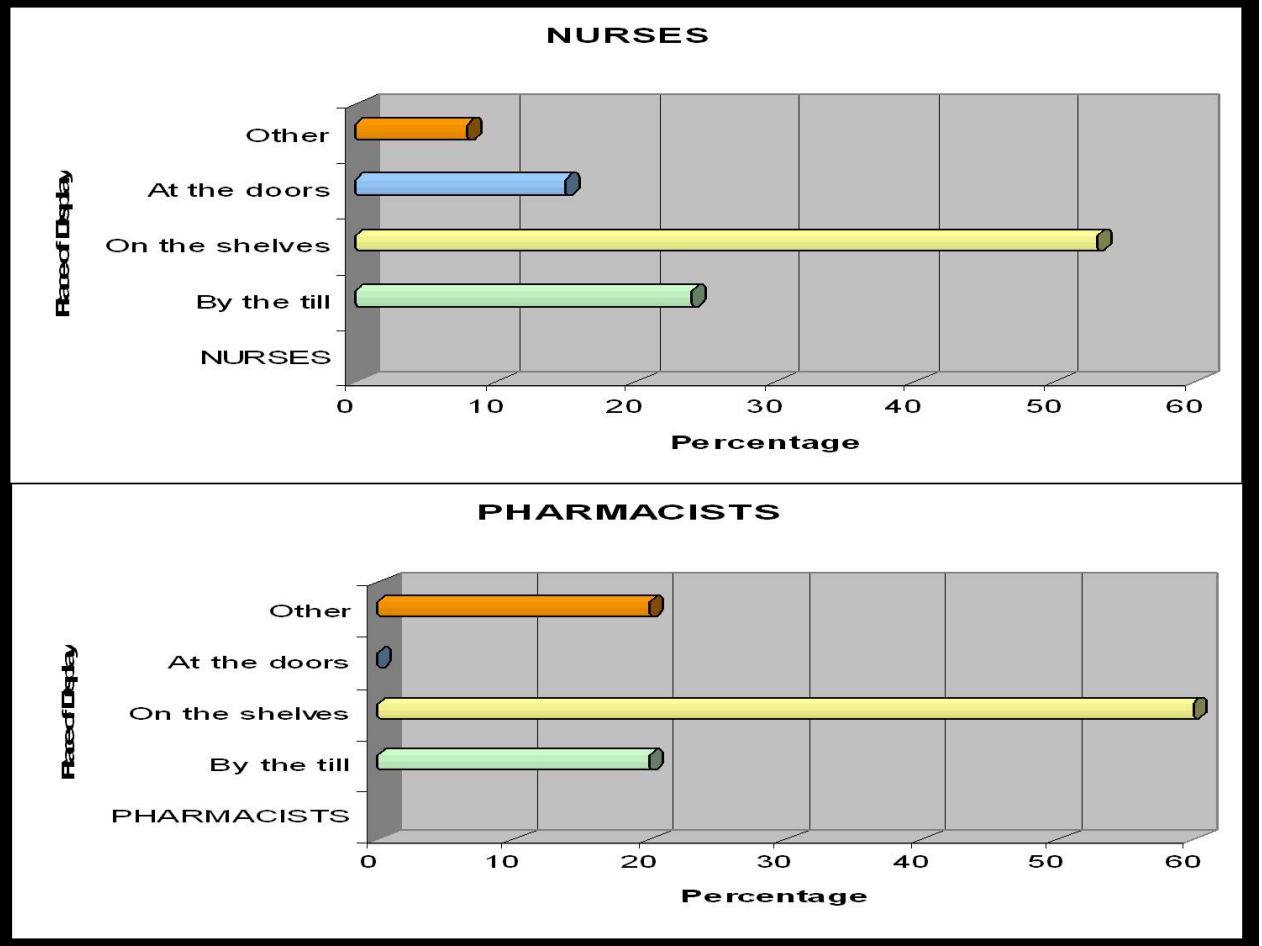
Preferences regarding the display of microbicides in stores among nurses and pharmacists.

Figure 3 indicates that the majority of HM's, pharmacists and nurses preferred microbicides to be displayed on the shelves in retail stores. The HCPs saw this as a way to facilitate access to the product and instructions (e.g., '*Because you can take your time and read the information about it on a box'*); and decrease discomfort (e.g., '*Some people may not be comfortable being seen and asking about the product. Shelves are private') *One pharmacist suggested that microbicides be displayed '*near the dispensary area' *to legitimize it as being health-related and allow greater privacy.

A nurse who favoured the display of microbicides at the till thought that '*everybody can... see them when they are standing in the queue' *while another nurse noted that that a person might be motivated to pick up this product while they are waiting.

#### Packaging

HCPs acknowledged that the '*packaging must appeal' *to potential users. Some suggested that microbicides be packaged in a box, while others preferred tubes for safety (e.g., '*to prevent it from any other contamination in the atmosphere'*. Some pharmacists advocated for user information and instruction leaflets and a clearly demarcated expiry date. Other pharmacists considered size (e.g., '*pack of tampons'*) and, material ('*cellophane containers like those used for cooler boxes...for the protection of the contents'*), and environment-friendly issues.

Nurses also suggested that '*the box should be small and sexy to fit a pocket or a purse and a moisture-proof container' *to prolong the expiry date.

#### Cost

Seventy percent (7/10) of the pharmacists and 86% (107/125) of nurses felt that microbicides should be provided free-of-charge so that '*all people will have access to them'*. Nurses also indicated that many people were unemployed and thus would be unable to afford microbicides unless they were free. The remaining 30% (3/10) of pharmacists felt that microbicides should be available both free-of-charge and for a fee – '*It can be both ways. For instance, condoms are free in clinics and you can buy them at chemists for those who can afford *[them]'. One nurse who preferred patients to pay for microbicides was concerned that free microbicides would '*encourage irresponsible *[sexual]*behaviour'*.

HCPs were willing to pay as little as R1 to as much as R50 (about $0.17 to $8.33) for the product. R5 – R20 ($0.80 to $3.33) seemed to be an acceptable amount for most participants. One pharmacist felt that a free trial period could be beneficial. Microbicides should be free *'for the first two years because this is marketing. Scientists must commit *[by]*giving to our people. By so doing, they'll be making the product desirable to the community'*.

## Discussion

HCPs who were primarily from the public sector serve most of the country. Therefore, their opinions on microbicides are of immense value. Approximately one third overall of HCPs were aware of microbicides. However, the depth of their knowledge varied. Whereas some had a good understanding of what microbicides are and their mechanisms of protection, others only knew that such products are being evaluated in research studies. Most knowledge of microbicides was acquired recently, indicating the power of information dissemination through the MRC and other health professionals, and perhaps mounting support. Given that the first results of phase III trials for some candidates are expected in late 2008, it is imperative that as many HCPs as possible are educated about microbicides now so that when available, they will be familiar with and perhaps more open to the concept. As front-line providers, they are well-positioned to impart accurate information to their clients. Since clients often take HCPs into their confidence, they can play a valuable role in uncovering and challenging misconceptions about microbicides, e.g., that microbicide testing will involve deliberately exposing participants to HIV. Addressing these speculative myths now will help to build confidence and trust in the effectiveness of microbicides when they become available.

It was extremely encouraging that the vast majority of HCPs were positive about microbicides, and many felt that they should ideally be used in conjunction with condoms. HCPs recognized the advantages that microbicides would have over condoms, especially for women, and nurses in particular described the lack of condom use among their clients.

Nearly four-fifths of all study participants felt that microbicides should be made available to everyone, regardless of age, HIV status, and history of sexually transmitted infections. HCPs recognize that they can no longer afford to restrict HIV prevention methods to certain groups because the entire population in South Africa is vulnerable, especially since rape and migrant labour contribute to the spread of HIV and AIDS. Nevertheless, some HCPs supported the restriction of access to microbicides due to concern that they would be seen as a "magic bullet" or cure, thus giving users a false sense of safety and perhaps license to engage in unprotected sex – an emergent concern with post-exposure prophylaxis [11]. In particular, restricted access to youth was noted.

The majority of HCPs reported that they would support microbicides if they are as good as condoms in preventing HIV transmission, especially because of the advantages that microbicides have over condoms. HCPs in this study were primarily concerned with the safety and size of the product applicator. If they are less effective, most pharmacists and nurses reported that they would be more likely to support microbicides for dual method use, since a partially effective microbicide would still provide another prevention option for women [12]. A method that is less effective, but used consistently, may have a greater impact on reducing infection than a higher-effectiveness method used less consistently. In fact, mathematical modelling indicates that a microbicide which is 40% less effective than condoms, but is used only by 30% of the population, will save 6 million lives the world over in three years [13]. HCPs should be informed of this fact as consistent use of future microbicides could be the key to decreasing the spread of HIV in South Africa.

Nurses, especially those in Hlabisa, demonstrated the significant effect that incorrect understanding can have on the perceptions of a product. A large number of them understood 'less effective' microbicides to be completely ineffective and as a result, did not want to recommend them to their clients. When such a product does become available, health care workers will need to be sufficiently trained regarding issues such as partial efficacy to prevent such errors. HCPs will need adequate information so that they are equipped to counsel patients in making informed choices.

The creation and dissemination of messages about microbicides now to HCPs will raise awareness of the potential of microbicides in reducing women's vulnerability to HIV and AIDS. In addition, early education will help to instill positive attitudes so that when an effective product comes to market, they will be prepared to counsel clients into using them. Since we anticipate that first-generation microbicides will likely have lower efficacy than condoms, we should begin to design hierarchical prevention messages for HCPs to incorporate into client counseling, as well as testing their complexity and appeal.

Overall support for microbicides from HCPs was overwhelming and correlates with data reported in other studies [14]. HCPs' dedication and sincere concern came across clearly in their willingness to counsel clients about microbicides and recommend the new products to others.

HCPs believed that the challenges facing the introduction of microbicides are extensive. Fewer HMs than nurses and pharmacists predicted barriers to the introduction of microbicides. This is significant because nurses and pharmacists are the ones who interact with patients and bring their issues across; therefore, their responses are probably a more valid reflection than those of the HMs. The obstacles foreseen ranged from cultural and religious beliefs to practical aspects, such as cost and characteristics of microbicides. Cultural practices are complex issues, and those such as the preference for dry sex and the importance of preserving a woman's virginity, will need to be addressed. The fact that microbicides will be novel was also raised, indicating that education of potential users will be necessary even prior to microbicides becoming available. Although health care facilities may be perceived as adequately prepared to handle the introduction of microbicides, additional staff resources will be required to address foreseeable shortages. Different health care centres will have their own challenges in rolling out the product and these will have to be dealt with on an individual basis. The relevance of these barriers mentioned by HCPs is that each challenge has the potential to halt the progress and use of microbicides. It is imperative, therefore, that plans are developed to overcome them, before the product is marketed for use.

Nurses and pharmacists were of the opinion that multiple organizations, such as hospitals, NGOs, other community organizations, and schools need to play a pivotal role in educating people about microbicides. Promotion of microbicides should take all forms. While the media was favoured as an effective marketing strategy, the written word was also perceived to be a credible source for delivering health messages. Thus, both print and non-print media should be used to publicize microbicides.

Further issues involving marketing revealed that HCPs favour over-the-counter sales of microbicides at pharmacies. This was to prevent added costs of consultation fees and so that patients will receive some counseling on the product. Adequate reasons were also provided on the preference of acquiring the product *via *a doctor's prescription or on the shelves. The same was noted for retail in stores – while the majority supported the display of the product on the shelves, others wished for them to be sold at the till. This indicates that multiple points of sale are probably the best option, but issues such as privacy, counseling and easy access need to be considered. Participants in a national US study reported that the ideal microbicide should be available in pharmacies without prescription and wanted someone to assist them in how to use the product (Darroch & Frost, 1999). However, most participants preferred microbicides to be free-of-charge; although some were concerned that free products would be perceived to be of inferior quality, which has been noted about free distribution of male condoms.

### Limitations

There were a number of limitations to the current study. Firstly, the majority of the study participants were women, reflecting the gender of most HCPs in South Africa's public health sector. Thus, whether male HCPs' beliefs and attitudes about microbicides differ from those of their female counterparts cannot be determined in this study. Study participants were self-selected volunteers, but selection bias was minimized as only a few of those approached refused to be interviewed individually or *via *a focus group. HCPs' responses to microbicides were based on hypothetical products, and could differ once an effective microbicide is identified.

## Conclusion

This the first comprehensive study to explore HCPs knowledge and attitudes regarding microbicides. Our data illustrates important insights into HCPs' level of awareness and knowledge about microbicides and the potential challenges to be faced, in the event of their introduction. Understanding HCPs' preferences for marketing strategies will be invaluable to prepare microbicides for distribution and optimize their acceptability, uptake, and continued use. This information can also assist in short-term strategic planning for the crafting of appropriate prevention messages and identification of distribution channels and messengers for information dissemination. No single approach will be sufficient to reach the diversity of provider and potential user target audiences.

Even in the absence of an efficacious product, increasing awareness of microbicides and their potential benefits and limitations will keep HCPs abreast of the current status of microbicide research findings. At the same time, continual evaluation of HCPs' current concerns, and after a microbicide is on the market, can correct misconceptions and help to shape positive attitudes and community norms about the possibility of this new women's HIV protection product.

## List of abbreviations

HCP = Health Care Provider

HM = Hospital Manager

PU = Potential User

CPN = Chief Professional Nurse

FGD = Focus Group Discussion

SSI = Semi Structured Interview

HIV = Human Immuno-deficiency Virus

AIDS = Acquired Immune Deficiency Syndrome

STI = Sexually Transmitted Infection

## Competing interests

The author(s) declare that they have no competing interests.

## Authors' contributions

GR was the principal investigator of this study and wrote the grant, protocol and did the data analysis for the study with collaborations from NSM and JEM. NSM assisted in writing the paper, protocol writing, data analysis, provided training and managed the team. JEM worked on the study grant and protocol, as well as provided reviews and input into the paper. JM assisted with the review and writing of the paper. as well as coordinated the project, supervised the field team and did quality control on the data. JM also facilitated the data collection process, contacted the service providers and set appointmenets for the data collection. VG assisted with the data analysis, drafting of the paper and cleaning of the data sets.
